# Editorial: Probing the Ubiquitin Landscape

**DOI:** 10.3389/fchem.2020.00449

**Published:** 2020-05-20

**Authors:** Monique P. C. Mulder, Zhihao Zhuang, Lei Liu, Benedikt M. Kessler, Huib Ovaa

**Affiliations:** ^1^Department of Cell and Chemical Biology, Oncode Institute, Leiden University Medical Centre, Leiden, Netherlands; ^2^Department of Chemistry and Biochemistry, University of Delaware, Newark, DE, United States; ^3^Tsinghua-Peking Center for Life Sciences, MOE Key Laboratory of Bioorganic Phosphorus Chemistry & Chemical Biology, Center for Synthetic and Systems Biology, Department of Chemistry, Tsinghua University, Beijing, China; ^4^Nuffield Department of Medicine, University of Oxford, Oxford, United Kingdom

**Keywords:** ubiquitin(-like), activity-based probes, toolbox development, applications, inhibitors, proteomics

Since the pioneering work of Hershko, Ciechanover and their colleagues 40 years ago, our understanding of one of the most complex and widespread signaling networks in biology has evolved greatly through genetic, proteomic, biochemical, and cell biological studies (Kliza and Husnjak). In this Research Topic, we present a salient collection of original research, methods and review articles that cover novel, promising and recent trends in the ubiquitin(-like) field.

Protein ubiquitination is a powerful post-translational modulator (PTM) as it controls almost every process in cells. To accomplish this, various ubiquitin (Ub) modifications adopt distinct conformations, utilizing what is commonly referred to as the “Ub code”, leading to different cellular functions. Modification by ubiquitin of a target protein is tightly controlled by the action of hundreds of regulatory enzymes employed in specific combinations involving three main steps: activation (E1 enzymes), conjugation (E2 enzymes), and ligation (E3 enzymes). To counterbalance ubiquitination, it can be removed from substrate proteins by deubiquitinating enzymes (DUBs). Modification of a substrate protein can occur by a single Ub moiety on a single target lysine (monoubiquitination) or on multiple lysines (multi-monoubiquitination). Additionally, after a single Ub is transferred to the substrate protein, any of the eight amino groups of the initial substrate-conjugated ubiquitin (Lys6, Lys11, Lys27, Lys29, Lys33, Lys48, Lys63, Met1) can be modified with another Ub molecule, yielding polyubiquitin chains of variable linkage type, length, and configuration (homo- vs. heterotypic Ub chains). Though the functional significance of cellular Ub modifications, such as Lys48- and Lys63-linked polyUb chains, are largely known, the biological significance of other homotypic polyUb chains, collectively referred to as atypical Ub chains, is still far from being fully understood. While van Huizen and Kikkert comprehensively review the role of atypical ubiquitin chains in the regulation of antiviral innate immunity pathways, Dittmar and Winklhofer review a distinct type of ubiquitination—linear (Met1-linked) ubiquitination—a transient and spatially regulated modification, complexifying their detection and quantification.

Next to Ub, a vast number of ubiquitin-like (UbL) proteins (e.g., SUMO, Nedd8, ISG15, Ufm1, Fat10) can also be attached to proteins increasing the complexity and fine-tuning cellular responses even further. Keiten-Schmitz et al. review the role of SUMO chains in chromatin dynamics and genome stability networks, whereas Fernández et al. describe ISGylation as well as strategies targeting this PTM for therapeutic applications. Intriguingly, unlike most PTMs such as acetylation and phosphorylation, ubiquitin itself can be highly customized through further post-translational modification by other PTMs, thereby expanding the Ub code for distinct cellular outcomes which either alter the originally encrypted message or encode a completely new one. The cross-functionality introduced through additional post-translational modification of Ub molecules by UbL proteins, rendering hybrid Ub/UbL chains, is discussed by Perez Berrocal et al..

Considering the impact of ubiquitination on the regulation of a vast array of fundamental biological processes, dysregulation of this intrinsic process gives rise to numerous diseases ranging from autoimmunity, cancer, and neurodegenerative diseases such as Alzheimer and Huntington's disease (Reits and Sap). During the last few years defects in the ubiquitin-proteasome system (UPS)—a central player in protein quality control facilitating elimination of misfolded or otherwise aberrant proteins—including hampered E3 ligase activity, have been the focus of many studies. Understanding the mechanism of action, as well as identifying which substrates are regulated by a given E3 ligase could provide invaluable knowledge toward the development of therapeutic strategies. Garcia-Barcena et al. review the generation and usage of E3 mutants, thereby highlighting the complexity of this family of enzymes. As the E3 enzymes exhibit different substrate specificity, determining which E3 enzyme maps to which substrate is crucial for our understanding of this complex intrinsic network. Salas-Lloret et al. describe an improved TULIP2 methodology facilitating mass spectrometry toward the identification of E3-substrate networks.

While inhibition of the UPS has proven promising in the treatment of cancer, stimulation of the proteasome has been proposed as a potential therapeutic strategy for neurodegenerative disorders. In the pursuit for novel therapeutics and intervention points, robust assays and tools are key toward understanding and identification of specific components of the UPS. Franklin and Pruneda describe a new assay, UbiReal, that make use of fluorescence polarization to monitor all stages of Ub conjugation and deconjugation in real time, making it a candidate for High-throughput screens (HTS) of activity modulators. In addition, the general functional status of the UPS in cells can be examined using reporter substrates as discussed by Gierisch et al.. More recently technologies have been established that induce targeted protein degradation by chimeric small molecules as reviewed by Naito et al.. These technologies, such as Proteolysis Targeting Chimeras (PROTACs), hijack the cellular machinery for ubiquitination thereby subjecting the ubiquitinated proteins to proteasomal degradation. This promoted several drug development research programs as proteins which had previously been regarded as “undruggable” by traditional small molecule therapies can now be degraded by inducing selective intracellular proteolysis.

With an increased focus on the development of novel therapeutics of ubiquitin(-like) system components, characterization of their dynamics is imminent. Understanding the mechanisms of ubiquitin regulation requires the generation of antibodies or alternative reagents that detect ubiquitin in a site-specific manner. van Kruijsbergen et al. describe a strategy and the encountered challenges toward the development of site-specific ubiquitin antibodies. Together with advances in synthetic strategies for ubiquitin generation, enabling the development of a plethora of ubiquitin activity-based probes (ABPs) and assay reagents, the study of enzymes involved in the complex system of ubiquitination is now within reach. ABPs react covalently at the active site on the enzyme, and thus represent a powerful method to report on specific enzyme activity and to evaluate cellular and physiological enzyme dynamics and function. Taylor and McGouran present the developments made in the “traditional” ubiquitin based ABPs, whereas Conole et al. discuss recent developments in cell-permeable small molecule ABPs. With these advances in the ABP field, activity based protein profiling has emerged as a powerful technique to study these important enzymes as exemplified by the work of Pinto-Fernández et al.. Here, they combined advanced mass spectrometry technology with propargylic-based ubiquitin ABPs to reveal the proportion of active cellular DUBs adding another layer of information in addition to their endogenous expression levels.

Further understanding of the ubiquitin signaling pathway includes the knowhow on how different polyUb chains are recognized by interacting proteins. Often these interacting proteins contain a specific ubiquitin-binding domain (UBD) that bind specifically to polyUb chains, thereby rendering different cellular outcomes. Hameed et al. report on the synthesis of diUb chains, fully 15N-labeled on the distal (N-terminal) Ub and demonstrate their applicability for gaining insights into linkage-selective ubiquitin recognition of a unique UBD.

We believe that our Probing the Ubiquitin Landscape Research Topic demonstrates the multidisciplinary nature of research in the ubiquitin field. Advancement in the field is dependent on increased understanding through interconnected research strategies, thereby allowing development of therapeutics as exemplified in this body of work.

## Author Contributions

All authors listed have made a substantial, direct and intellectual contribution to the work, and approved it for publication.

## Conflict of Interest

The authors declare that the research was conducted in the absence of any commercial or financial relationships that could be construed as a potential conflict of interest.

